# A novel loop-head guidewire-assisted endoscopic retrograde appendicitis therapy for fecalith obstruction

**DOI:** 10.1055/a-2512-3754

**Published:** 2025-01-21

**Authors:** Mingwen Guo, Wenguang Yang, Yijuan Guo, Yuhong Ren, Bin Yang, Sichao Wen, Haiyong Long

**Affiliations:** 1 Department of Gastroenterology, Qionglai Medical Center Hospital, Qionglai, China

Acute appendicitis represents one of the most prevalent acute abdominal conditions, with appendiceal fecaliths being the primary etiological factor 1 . Endoscopic retrograde appendicitis treatment (ERAT) represents a novel approach for managing appendicitis and constitutes a micro-innovative technique aimed at alleviating appendiceal obstruction 2 . ERAT is a quick procedure and is associated with minimal trauma, swift recovery, low complication rate, no surgical scars, and maintains the immune function of the appendix 3 . Currently, upon accessing the appendiceal orifice via colonoscopy, there are typically two methods of entering the appendiceal cavity: one involves the use of a transparent cap to assist with zebra guidewire intubation, while the other employs an imaging catheter to navigate into the appendiceal cavity under direct visualization. Owing to the unique anatomical structure of the appendiceal cavity, both methods present significant challenges for access, frequently resulting in operational failure 4 .

We report the case of a 62-year-old woman who was admitted to our department with a diagnosis of appendicitis accompanied by a fecalith ( Fig. 1 **a** ). Colonoscopic examination revealed mild mucosal congestion and edema at the appendiceal orifice ( Fig. 1 **b** ). We utilized a self-made loop-head guidewire to intubate the appendiceal cavity ( Fig. 1 **c** ). The loop guidewire facilitated improved entry into the Gerlach flap and navigated the irregular appendiceal cavity more effectively, thereby reducing the complexity of intubation within the appendiceal cavity ( Video 1 ). The sphincterotome knife was introduced into the appendix alongside the guidewire for angiographic evaluation. Fluoroscopy revealed the presence of multiple filling defects within the appendix ( Fig. 1 **d** ). A calculus-removing balloon was employed to extract fecaliths from the appendiceal cavity under fluoroscopic guidance ( Fig. 1 **e** ). Some yellow fecaliths were visible during the colonoscopy ( Fig. 1 **f** ). The patient recovered smoothly and was discharged within 2 days post-procedure.

**Fig. 1:**
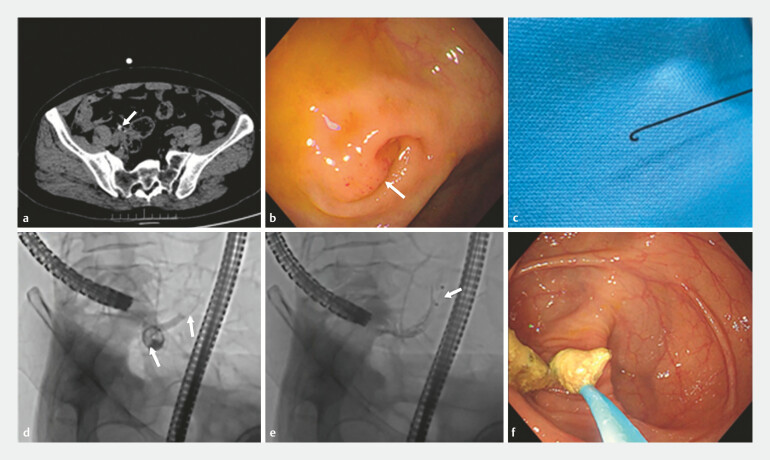
Novel loop-head guidewire-assisted endoscopic retrograde appendicitis therapy for fecalith obstruction. **a** An abdominal computed tomography scan showed appendicular fecaliths. **b** Mild mucosal congestion and edema at the appendiceal orifice. **c** Self-made loop guidewire. **d** Fluoroscopy showed multiple filling defects in the appendix. **e** A calculus-removing balloon was used to remove the fecaliths. **f** Many fecaliths were removed.

A novel loop-head guidewire-assisted method for endoscopic retrograde appendicitis therapy for fecalith obstruction.Video 1

The loop-head guidewire simplifies intubation of the appendiceal cavity, potentially increasing the success of ERAT and advancing the technology.

Endoscopy_UCTN_Code_TTT_1AQ_2AJ

## Notes

Endoscopy E-Videos https://eref.thieme.de/e-videos
*E-Videos* is an open access online section of the journal *Endoscopy* , reporting on interesting cases and new techniques in gastroenterological endoscopy. All papers include a high-quality video and are published with a Creative Commons CC-BY license. Endoscopy E-Videos qualify for HINARI discounts and waivers and eligibility is automatically checked during the submission process. We grant 100% waivers to articles whose corresponding authors are based in Group A countries and 50% waivers to those who are based in Group B countries as classified by Research4Life (see: https://www.research4life.org/access/eligibility/ ). This section has its own submission website at https://mc.manuscriptcentral.com/e-videos .
